# Supplementary figures and images for: Homeodomain protein Six4 prevents the generation of supernumerary Drosophila type II neuroblasts and premature differentiation of intermediate neural progenitors
Source: PLoS Genet. 2021 Feb 8;17(2):e1009371. doi: 10.1371/journal.pgen.1009371 (PMC7895384; doi:10.1371/journal.pgen.1009371)

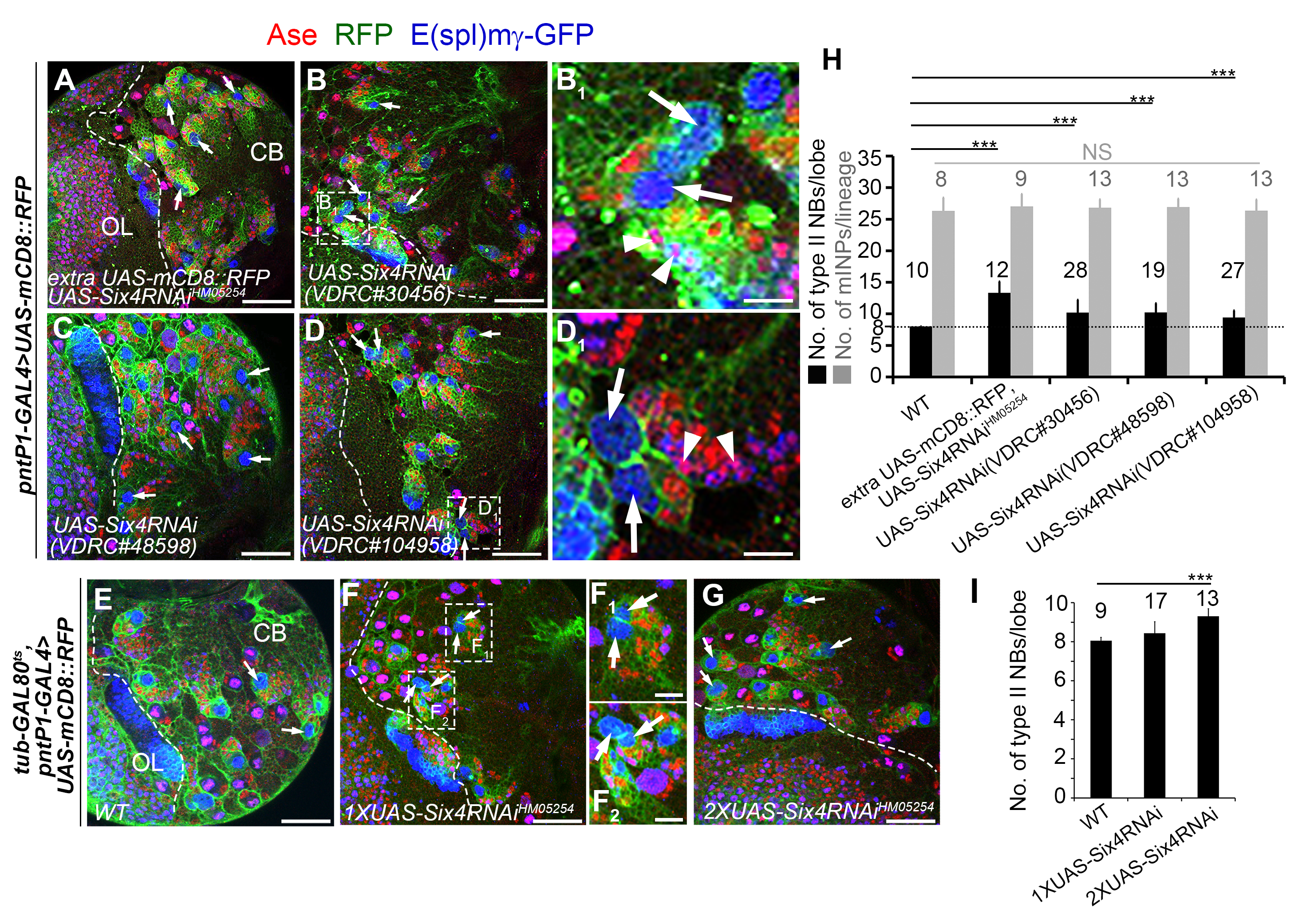

Supplement: S1 Fig — Type II NB lineages are labeled with mCD8-RFP (in green) driven by pntP1-GAL4 and counterstained with anti-GFP (in blue) and anti-Ase (in red) antibodies. Arrows point to type II NBs. Dashed lines demarcate the boundary between the central brain (CB) and the optic lobe (OL). Scale bars equal 50μm in (A-B, C-D, E-F and G) or 10μm in (B1, D1, F1 and F2). (A) The supernumerary type II NB phenotype resulting from the expression of UAS-Six4 RNAiHM05254 is not affected by expressing an additional copy of UAS-mCD8-RFP. (B-D1) Expression of three additional independent UAS-Six4 RNAi transgenes results in consistent supernumerary type II NBs. (B1) and (D1) are enlarged views of the areas highlighted with dashed squares in (B) and (D), respectively, showing a single isolated lineage contains two type II NBs (arrows). Arrowheads in (B1) and (D1) point to mINPs. (E) A wild type larval brain lobe contains only 8 type II NBs. (F-G) Larval-specific expression of one (F-F2) or two copies (G) of UAS-Six4 RNAi driven by pntP1-GAL4 in combination with tub-GAL80ts to larval stages still leads to the generation of extra type II NBs. (F1) and (F2) are enlarged views of the areas highlighted with dashed squares in (F). Note that two type II NBs (arrows) co-exist in a single lineage in both (F1) and (F2). (H) Quantifications of the number of type II NBs and mINPs in brains expressing independent UAS-Six4 RNAi transgenes. The number on top of each bar indicates the number of brains or lineages examined. ***, p < 0.001; NS, not significant. (I) Quantifications of the number of type II NBs in brains expressing one or two copies of UAS-Six4 RNAi after larval hatching. The number on top of each bar indicates the number of brains examined. ***, p < 0.001. (TIF) [file pgen.1009371.s001.tif]

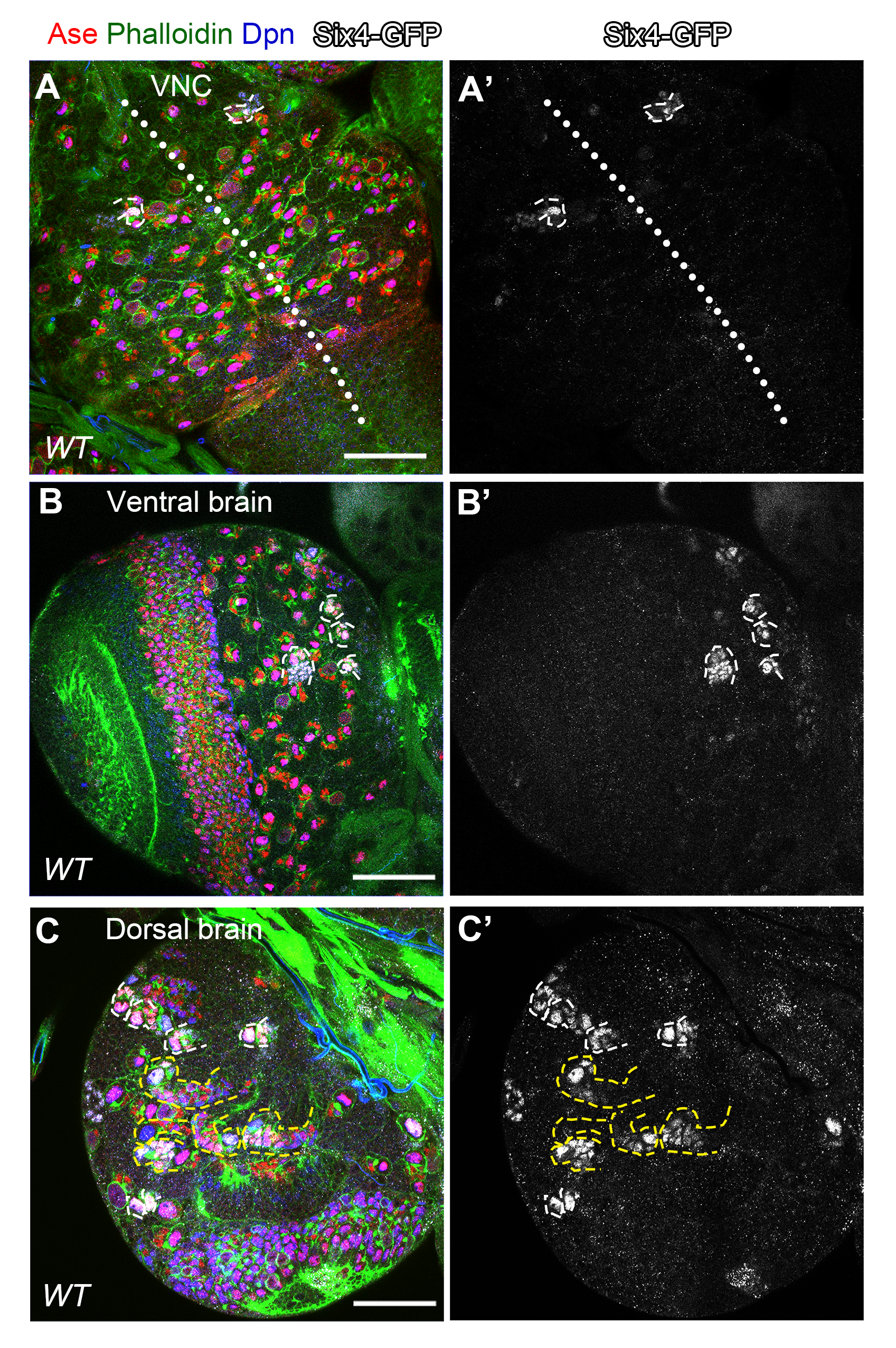

Supplement: S2 Fig — 3rd instar larval brains and VNCs are stained with Phalloidin to outline individual NB lineages and counterstained with Dpn and Ase antibodies for identifying type I and type I NBs. Scale bars equal 50μm. (A-A’) Six4-GFP is expressed in NBs and GMCs in two type I NB lineages in the VNC. Doted lines: the midline of the VNC. (B-C’) Several type I NB lineages (white dashed lines) in ventral (B-B’) and dorsal (C-C’) brain lobes have Six4-GFP expression in NBs and GMCs. Yellow dashed lines in (C-C’) outline type II NB lineages. (TIF) [file pgen.1009371.s002.tif]

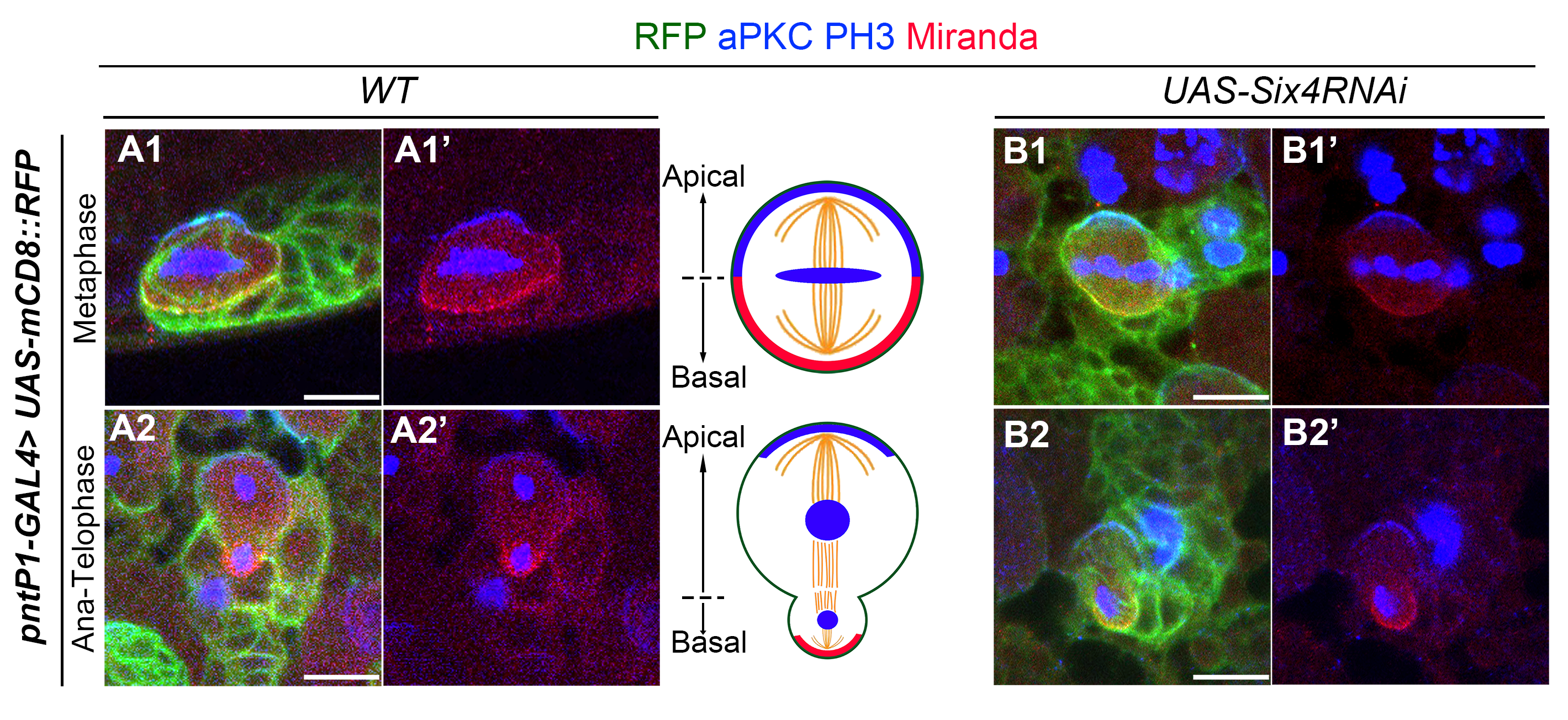

Supplement: S3 Fig — Type II NB lineages are labeled with mCD8-RFP driven by pntP1-GAL4. Mitotic marker pH3 staining is used to mark chromosomes. Scale bars equal 10μm. (A1-A2’) In wild type type II NBs, Mira (red) is segregated to the basal cortex and aPKC (blue) is segregated to the apical cortex at the metaphase (A1-A1’) and the anaphase/telophase (A2-A2’). The diagrams on the right show the distributions of Mira and aPKC at the metaphase and anaphase/telophase. (B1-B2’) In Six4 knockdown type II NBs, Mira and aPKC are segregated to the basal and apical cortex, respectively, at the metaphase (B1-B1’) and the anaphase/telophase (B2-B2’) as in the wild type type II NBs. (TIF) [file pgen.1009371.s003.tif]

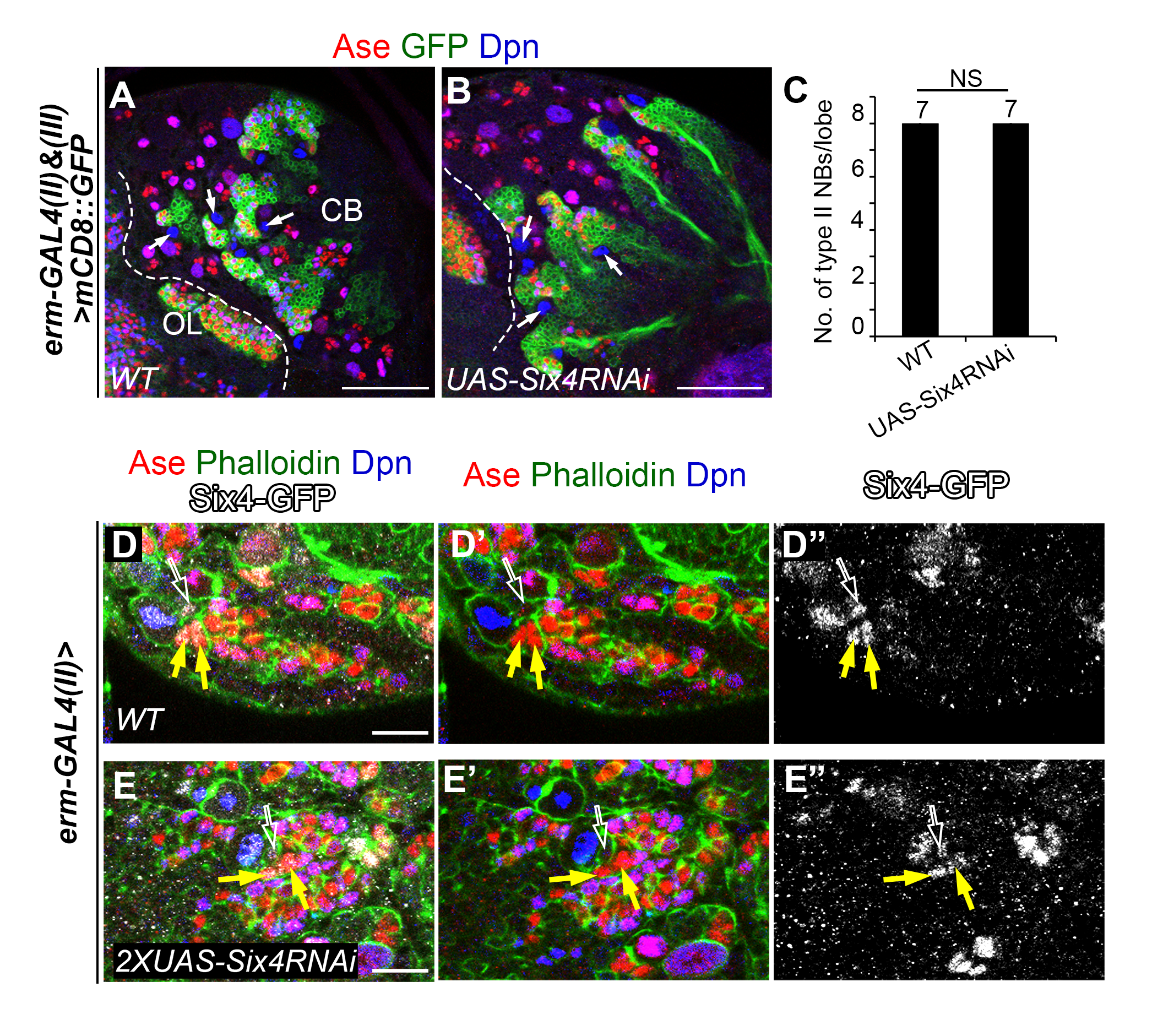

Supplement: S4 Fig — (A-C) A larval brain expressing UAS-Six4 RNAi driven by erm-GAL4(II) together with erm-GAL4(III) (B) has the same number of type II NBs as a wild type brain does (A). (C) Quantifications of the number of type II NBs in the wild type brains and brains expressing UAS-six4 RNAi driven by erm-GAL4 (II) and erm-GAL4(III). The number on top of each bar indicates the number of brains examined. NS, not significant. (D-E”) Six4-GFP expression in wild type type II NB lineages (D-D”) and type II NB lineages that express two copies of UAS- Six4 RNAi driven by erm-GAL4(II) (E-E”). Open arrows point to Ase- Dpn- imINPs and yellow arrows point to Ase+ Dpn- imINPs. (TIF) [file pgen.1009371.s004.tif]

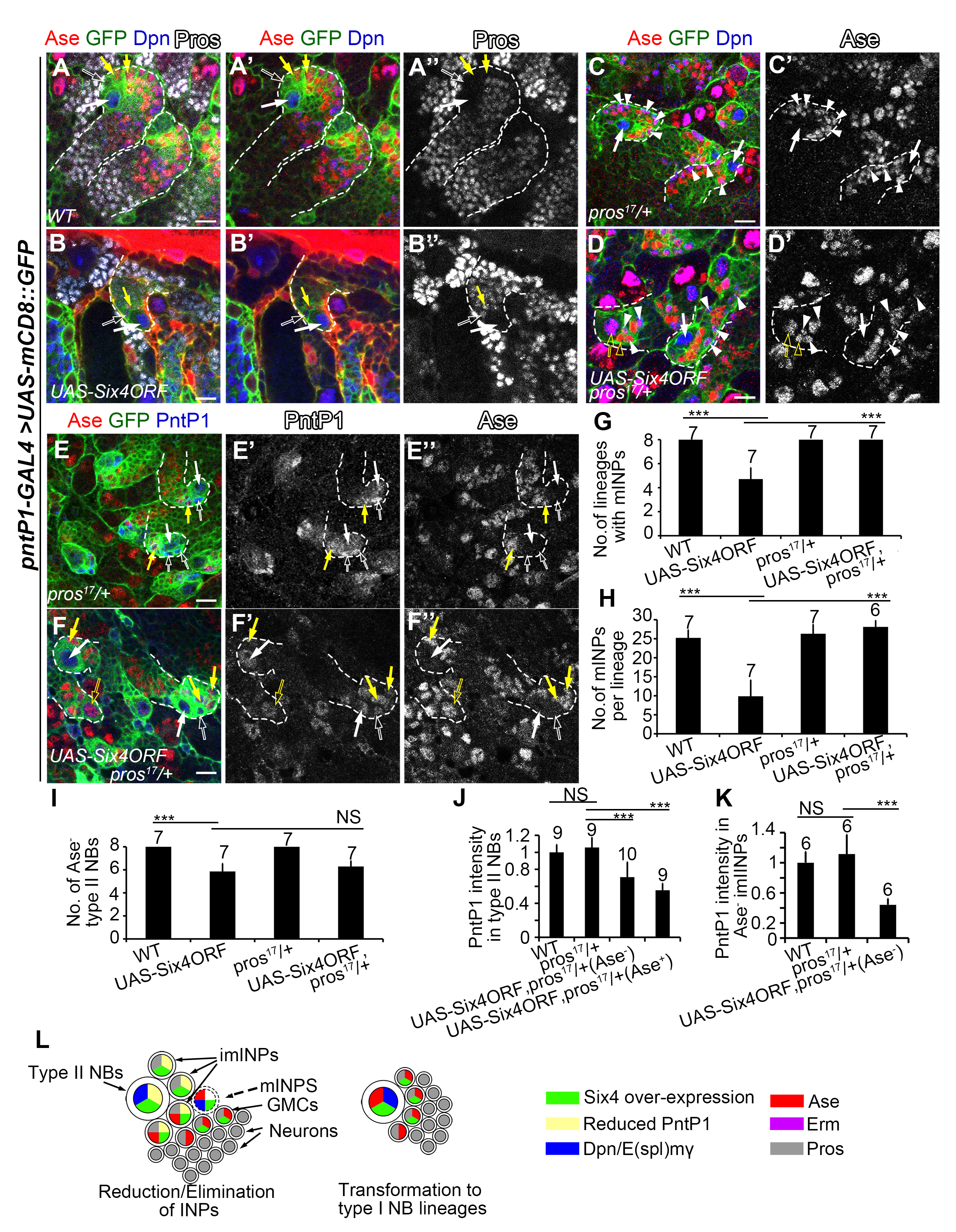

Supplement: S5 Fig — In all images, type II NB lineages are labeled with mCD8-GFP driven by pntP1-GAL4 and counterstained with antibodies against Dpn, Ase, Pros and/or PntP1. Type II NB lineages are outlined by dashed lines. Scale bars equal 10μm. (A-A”) In wild type type II NB lineages (dashed lines), nuclear Pros is not detected in either Ase- (open white arrows) or Ase+ (yellow arrows) imINPs. White arrows point to type II NBs. (B-B”) Nuclear Pros is ectopically expressed in Ase- (open white arrows) and Ase+ imINPs (yellow arrows) when Six4 is overexpressed in type II NBs. Note that Dpn+ mINPs are eliminated in the lineage outlined by the dashed line. (C-C’) pros17/+ mutant type II NB lineages have normal mINPs (white arrowheads) associated with Ase- type II NBs (white arrows). (D-D’) mINPs (white arrowheads) are largely rescued in all lineages when Six4 is overexpressed in pros17/+ heterozygous mutant type II NB lineages even though Ase is still ectopically expressed in a subset of type II NBs (open yellow arrows) and their newly generated imINPs (open yellow arrowheads). White arrows point to type II NBs without the ectopic Ase expression. (E-E”) pros17/+ mutant type II NBs and imINPs have normal PntP1 expression in type II NBs (white arrows) and Ase- (open white arrows) and Ase+ (yellow arrows) imINPs. Note that imINPs, particularly the Ase- imINPs, usually have much higher expression of PntP1 than type II NBs. (F-F”) When Six4 is overexpressed in pros17/+ mutants, PntP1 expression is partially still reduced in both Ase+ type II NBs (open yellow arrows) and Ase- type II NBs (white arrows) although the expression of PntP1 in the Ase+ type II NBs is partially restored. The expression of PntP1 in Ase- imINPs (open white arrows) and Ase+ imINPs (yellow arrows) is also reduced to levels that is comparable to or even lower than that in the type II NBs in the same lineages. (G-I) Quantifications of the number of type II NB lineages with mINPs (G), number of mINPs per lineage (H), and num [file pgen.1009371.s005.tif]

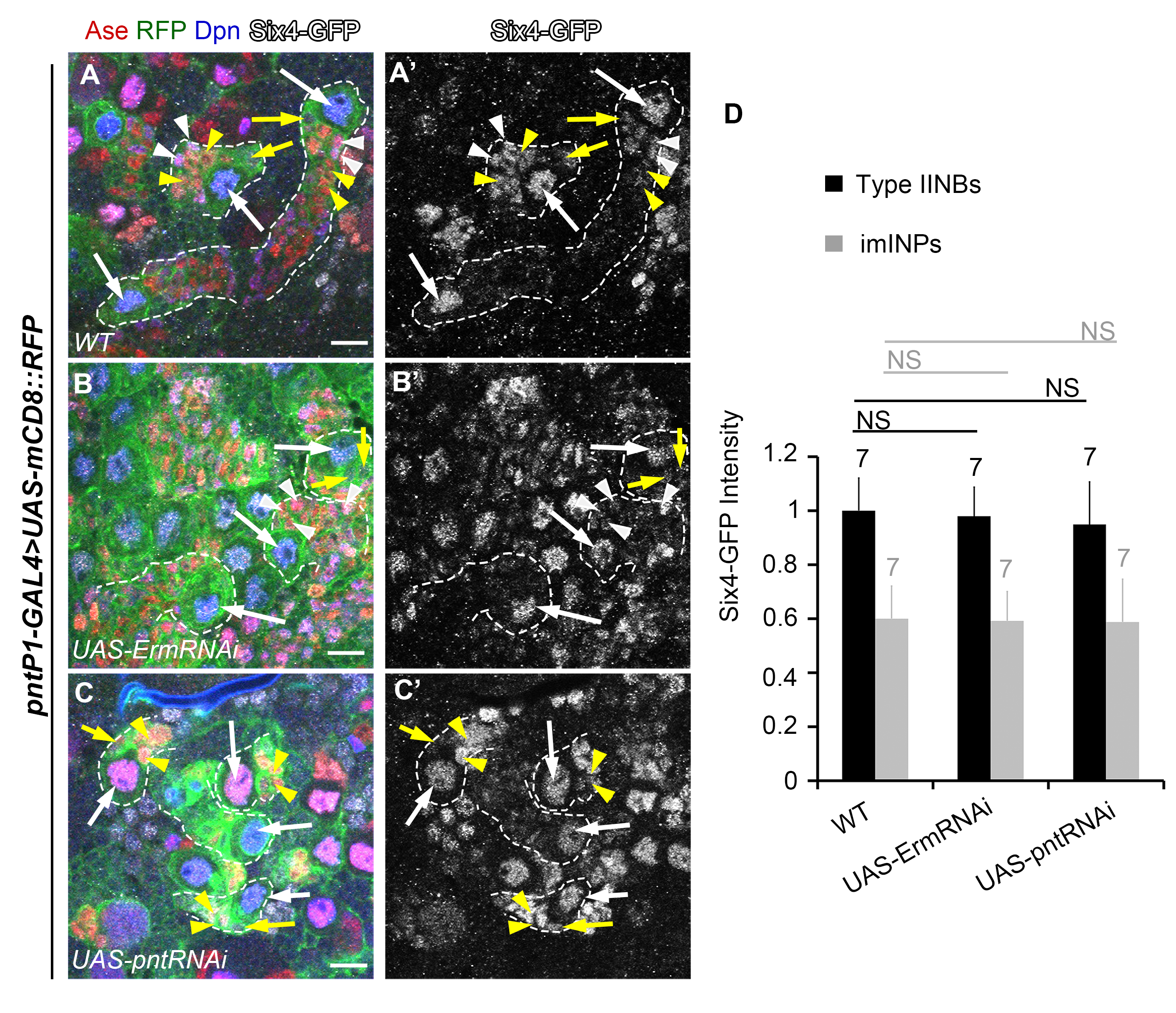

Supplement: S6 Fig — (A-C’) Similar Six4-GFP expression levels in type II NBs, imINPs, and mINPs are observed in wild type (A-A’), Erm knockdown (B-B’), and PntP1 knockdown (C-C’) type II NB lineages. Type II NBs lineages (outlined by dashed lines) are labeled by mCD8-RFP driven by pntP1-GAL4 and counterstained with antibodies against Dpn, Ase, and GFP. White arrows, type II NBs; yellow arrows, imINPs; white arrowheads, mINPs; yellow arrowheads, GMCs. Scale bars equal 10μm. (D) Quantifications of Six4-GFP staining intensity in type II NBs or Ase- imINPs in wild type, Erm knockdown, and PntP1 knockdown type II NB lineages. NS, not significant. (TIF) [file pgen.1009371.s006.tif]

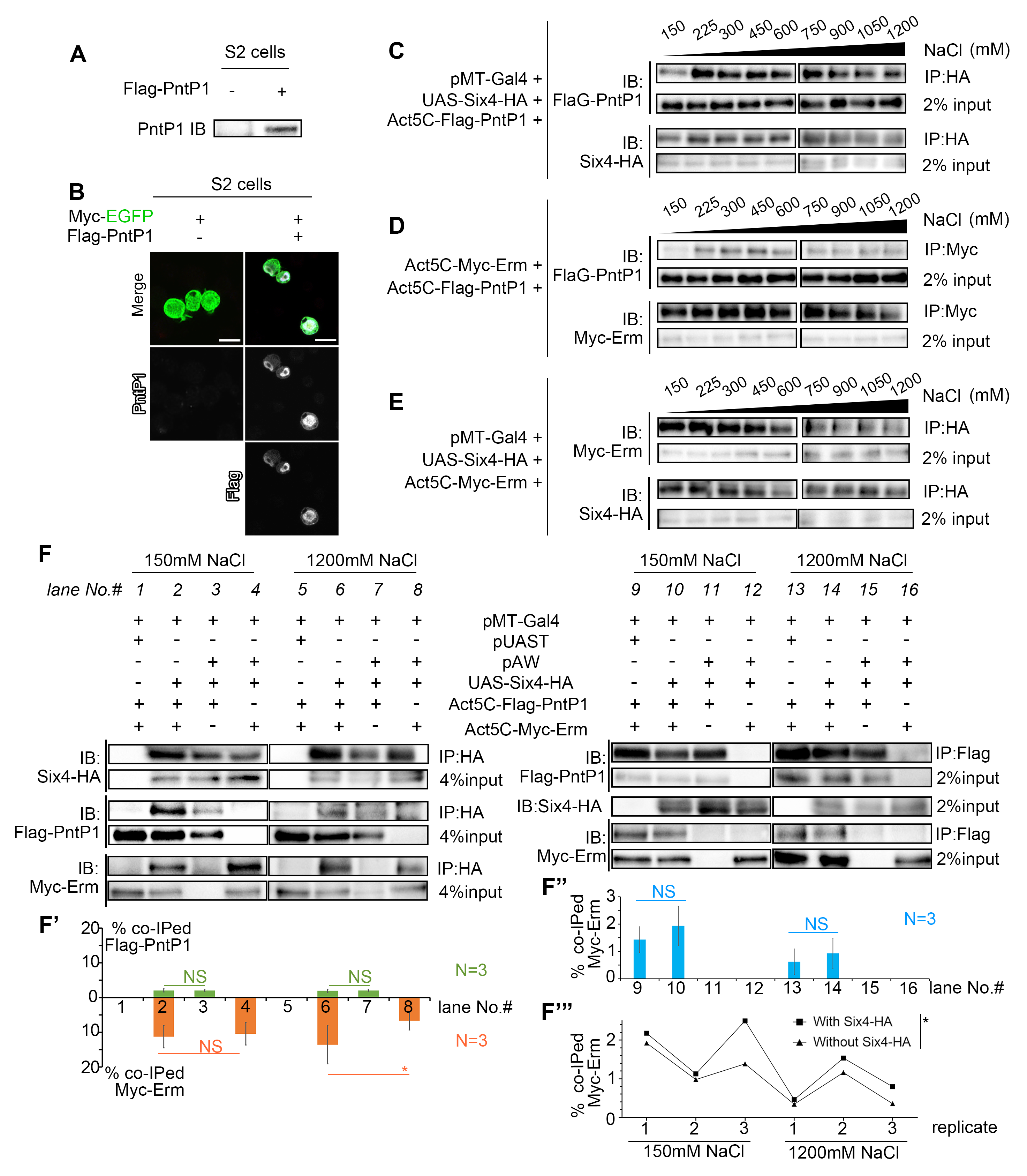

Supplement: S7 Fig — (A-B) Transfected Flag-PntP1 but not endogenous PntP1 are detected by western blot (A) or immunostaining (B) in S2 cells using the anti-PntP1 antibody or anti-Flag antibody. S2 cells are labeled with transfected Act5c-Myc-EGFP and scale bars equal 10μm in (B). (C-E) Co-IP of Six4-HA and Flag-PntP1 (C), Myc-Erm and Flag-PntP1 (D), and Six4-HA and Myc-Erm (E) from S2 cells with graded concentrations (150mM– 1200mM) of NaCl. Note that interactions between these protein pairs can still be observed but are compromised at higher concentrations of salt with the interaction between Six4-HA and Myc-Erm being compromised most. (F) Co-IP of Six4-HA, Flag-PntP1, or Myc-Erm by the anti-HA antibody (lanes #1–8) or anti-Flag antibody (lanes #9–16) with 150mM or 1200mM of NaCl from S2 cells that express a combination of any two or all three of these proteins. Note that when all these three proteins are expressed together in S2 cells, they can be pulled down in the same complex (lanes #2, #6, #10, and #14). Because of unusually high background due to unknown reasons, the bands for co-IPed Six4-HA with the anti-Flag antibody were not shown, but the 2% input shows that Six4-HA was indeed expressed when its expression DNA construct was transfected into S2 cells. (F’-F”) Quantifications of normalized % co-IPed Flag-PntP1 or % co-IPed Myc-Erm by the anti-HA (F’) or anti-Flag (F”) antibody in the indicated lanes in panel (F). The % co-IP was calculated by dividing the co-IPed protein by the 100% input of the co-IPed protein, then normalized by the IPed protein to correct the differences in immunoprecipitation. Note that the % co-IPed Myc-Erm by the anti-HA antibody with 1200mM of NaCL is significantly increased when Flag-PntP1 is co-expressed in S2 cells (compare lane #6 with lane #8). The % co-IPed Erm by the anti-Flag antibody with both 150mM and 1200 mM of NaCl is also consistently increased although not statistically significant when Six4-HA is co-expressed (compare lane #10 with lane [file pgen.1009371.s007.tif]

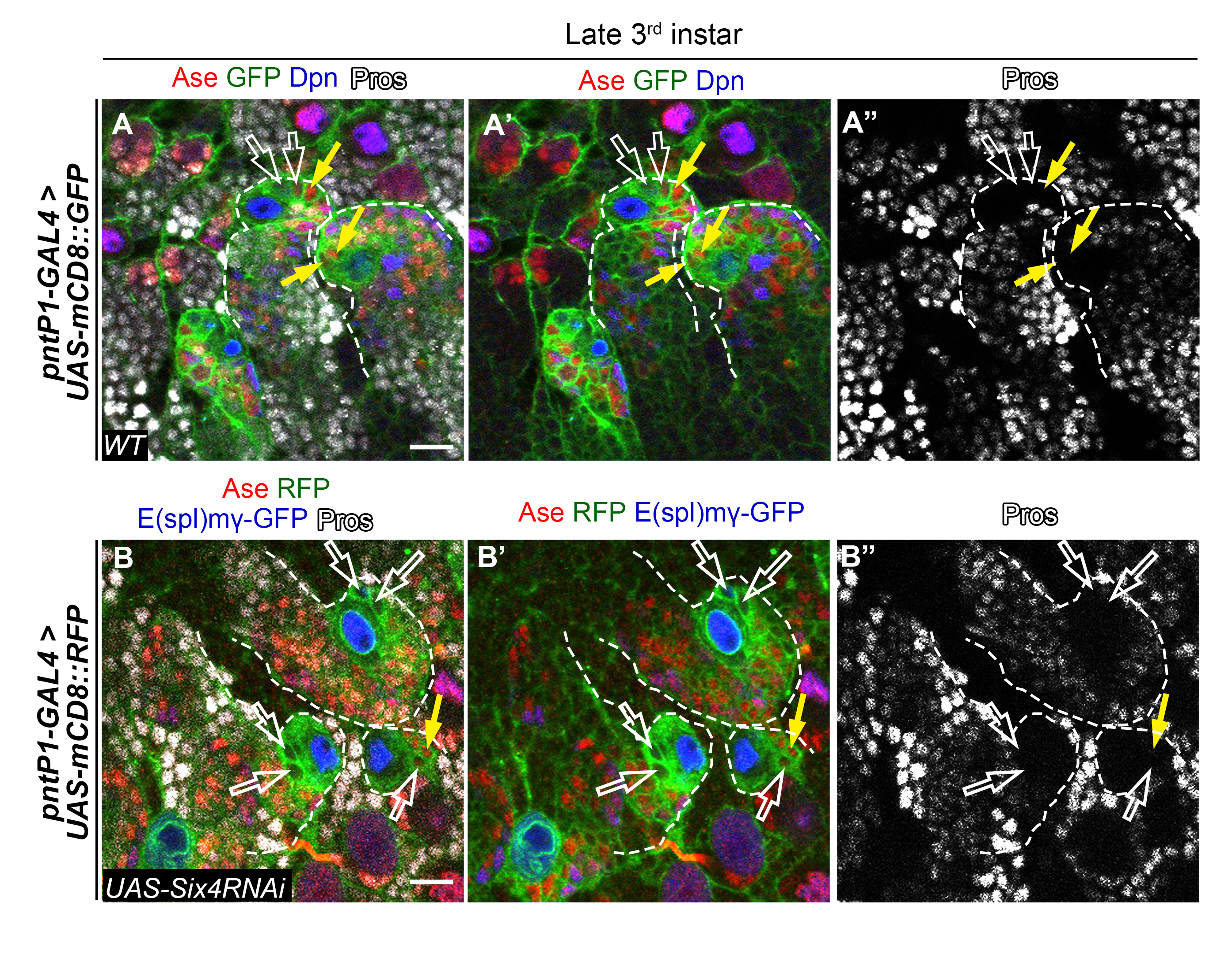

Supplement: S8 Fig — Nuclear Pros is not detected in either Ase- (open white arrows) or Ase+ (yellow arrows) imINPs in both wide type (A-A”) or Six4 knockdown (B-B”) type II NB lineages. Type II NB lineages are labeled with mCD8-GFP or mCD8-RFP driven by pntP1-GAL4 and outlined with dashed lines. Scale bars equal 10μm. (TIF) [file pgen.1009371.s008.tif]
