# Supplementary material for: Homeodomain protein Six4 prevents the generation of supernumerary Drosophila type II neuroblasts and premature differentiation of intermediate neural progenitors
Source: PLoS Genet. 2021 Feb 8;17(2):e1009371. doi: 10.1371/journal.pgen.1009371 (PMC7895384; doi:10.1371/journal.pgen.1009371)
Supplement: Attachment — Submitted filename: PloS genetics reviews -point by point response_final.docx [file pgen.1009371.s010.docx]

Reviewer #1: A prominent mechanism of expanding the pool of neural progenitors in the fruit fly brain is the generation of intermediate progenitors by neuroblasts. In the central brain there are several neural lineages (termed type II NBs) that display such a way of proliferation. While much is known on the switch from NB to INP, the mechanism that maintain INPs in their state remain less understood.

The authors show first that Six4 RNAi in type II NB lineages (under Pnt-Gal4) increases the number of NBs. Next they show that Six4 is highly expressed in type II NBs and that expression decreases in INPs. They provide evidence that Six4 knock-down does not affect asymmetric cell division. A set of genetic experiments using specific genetic markers (e.g. Erm, PntP1, BTD and Pros for early vs late INP) further supports that the Six4 and Erm are critical for early/immature INPs by regulating PntP1- the authors provide support that this is likely a direct action.

In summary I enjoyed reading the manuscript, it’s an intriguing addition to the type II mode of neurogenesis. The data are overall well-presented and of high quality. I only have a couple comments that were unclear to me.


>I do see the mechanisms of Six4 and Erm to act within the lineage, but I am not sure how an early defect of splitting or duplicating lineages can be excluded? Among these lines in principle the additional NBs should be observed within a lineage (fig1), however it is unclear to me where individual lineages have their boundaries and where extra NBs are localized.

Response: We have replaced with images (now as new Fig 1C and G, I) to show that multiple type II NBs could be observed in about 4% of single Six4 knockdown type II NB lineages at the 3^rd^ instar larval stages. Furthermore, we added new data in Fig. S1A-B to show that supernumerary type II NBs were still generated even if the expression of UAS-Six4 RNAi was turned on only after larval hatching, although the phenotype was relatively weak. Therefore, together with the results from Six4 knockdown with erm-GAL4 in imINPs, our data suggest the supernumerary type II NBs is likely generated through dedifferentiation of imINPs rather than due to an early defects of splitting or duplicating lineages. The reason most supernumerary type II NBs establish their own independent lineages could be because the dedifferentiation of imINPs happens at a low frequency when Six4 was knockdown.

We realized that the image quality was very low in the automatically converted PDF file. We tried many times with different approaches, but we were not able to improve the image quality in the automatically converted PDF file. Unfortunately, we are not allowed to upload our own PDF file, which will have much better image quality. So, to view high resolution images, please download the individually uploaded figures. We are sorry for the inconvenience.
> the main point of Figure 2 is to show Six4 expression, however while I agree that a triple/quadruple staining is informative, the way the wildtype data is represented is a bit confusing. Some image without the linage-depiction lines should be provided.

Response: we have removed dashed lines and yellow arrow/white arrowheads for some of the lineages in new Fig 2. And we left white arrows to point to type II NBs. In addition, new insets now show just one enlarged type II NB lineage instead of two so that the expression of Six4 in different cell types in type II NB lineages can be much better visualized.

Some expression remains in the RNAi knock-down. It would be helpful if the authors could comment on this.

Response: we really did not see any real Six-GFP signal remaining in Six4 knockdown type II NB lineages. We believe that what the reviewer referred to as “remaining signal” is just some background, which can be visualized throughout the entire brain lobe.

Reviewer #2: The manuscript by Rui Chen and colleagues addresses a very interesting question in neural development; namely, the mechanisms by which neural stem cells regulate the ultimate number of neurons and glial cells that are produced. These cells must prevent themselves from remaining stem cells to long but at the same time must prevent them from differentiating too quickly. When I read the abstract and introduction, I was very excited about his paper. The topic is very interesting and the authors had proposed a very nice mechanism.

However, I have significant concerns about the results section. I had a very hard time matching the text of the results with the figures.

1. My most significant issue is that had the authors had not placed arrows on each panel I would have not been able to see the results that they described in the text. I personally think that phenotypes should be displayed in an unbiased manner for the reviewer to evaluate. I don’t think our attention should be drawn to any particular region of an image. This paper is pretty egregious example – each panel with the main figures are littered with arrows – Figure 6K is a good example of what I am talking about. I tried to imaging what the figures would look like without the arrows. I concluded that I would not have been able to see what the authors are describing.

Response: we have removed most of the arrows or arrowheads in most figures, leaving only a few arrows/arrowheads pointing to a small subset of type II NBs lineages, except that in Fig 3. We think that in Fig 3 the arrows help readers to locate the type II NBs, considering that the phenotype is relatively weak. To make it easier to distinguish between type I and type II NBs, we also switched colors in all figures so that Dpn/E(spl)mg-GFP appear as blue and Ase as red such that all type II NBs appear in blue and type I NBs and INPs in magenta.

2. In most of the images, the authors have placed arrows on cells that look identical to neighboring cells that do not have arrows. Thus it is difficult for me to agree with many of the conclusions in the text.

Response: As stated above, we have switched the colors for Dpn/E(Spl)mγ-GFP/Ase staining to make it easier to distinguish type II NBs from type I NBs or INPs. We also removed an excessive number of arrows/arrowheads. We hope that the reviewers will find it easier now to see the differences among different cell types.

3. The figures (at least the version that I downloaded) are of pretty poor quality. In many cases, I cannot even use the arrows as a guide. It looks like they are not pointing to any particular cell type.

Response: We apologize for the poor quality of images in the automatically converted PDF file. We tried many times with different approaches, but the image quality in the automatically converted PDF file was always very low. However, all figures that we uploaded separately had a high resolution of 400dpi. In the automatically converted pdf file, there was a hyperlink for each figure for downloading the original Tif files. Unfortunately, we are not allowed to upload our own PDF file, which will have much better image quality. Therefore, to view the high resolution figures, please download the individually uploaded figures.

4. There are some quantifications that do not match the figures. For example, in figure 3 the expression of 2 copies of UAS-Six4 RNAi using erm-GAL4 is supposed to generate 1-2 additional type II NBs – however in figure 3B the authors are pointing to the same number of type II NBs as there are in the wild type shown in figure 3A. The chart in figure 3E indicates that there are 9 type II NBs on average in the mutant genotype.

Response: We are sorry that we did not make it very clear that in the original Fig 3B (now Fig 3C in the revised manuscript), two additional type II NBs from different focal slices were shown in the two insets (therefore there were total 10 type II NBs in this particular brain lobe). In the revised Fig 3C, we indicated the regions where the images shown in the two insets were imaged from.

5. I think images need to be presented in a completely unbiased manner. Use arrows sparingly and allow the reviewer to make up his or her own mind without guidance on the panels. You might need to provide higher magnification images as many of the cells that you are pointing too are too small to us to analyze. And you might consider altering the color combinations to make your results easier to see. The differences between magenta and red cells is sometimes not clear in the present images.

Response: We appreciate the suggestion from this reviewer. As stated above, we have removed most arrows/arrowheads in most figures. We also switched colors to make type II NBs appears as blue and type I NBs as magenta as the reviewer suggested to make it easier to distinguish between type I and type II NBs. Also, we ensure that all the figures uploaded separately have a high resolution of 400dpi.

6. You might also consider generating a new figure (figure 1) in which you provide the readers with a set of schematics that describe the neuroanatomy of the brain – something akin to figure 3G. Start with a zoomed out view of the entire brain and make a series of successive schematics that progressively zoom into the area of the brain that we will be looking at in each panel.

Response: Thank for the reviewer’s suggestion. We have added a diagram in Fig 1A to show the anatomy of Drosophila larval brains, with characteristic localization of two different types of NBs, lineage schemes, typical molecular markers, and the area we focused on for imaging.

Reviewer #3: In this manuscript Chen et al. explore the mechanisms involved in the integrity and homeostasis of intermediate neural precursors (INPs). For that, they use a multitude of genetic and biochemical tools to disclose the mechanisms regulating fate-fidelity in neural stem cells and progenitor cells, using Drosophila’s neuroblasts (NBs) and immature precursor progenitors (INPs) as their model. In order to identify genes that are necessary for proper INP development, the authors conducted a screen where they specifically abolish the levels of transcription factors within type II NBs and then described how it affected proper lineage formation, in terms of number of NB, immature INPs (imINPs) and mature INPs (mINPs) within the central brain lobes. From this screen, they identified that downregulation of Six4 (that is consistently and more strongly expressed in type II NB, with progressive but consistent expression throughout the lineage) resulted in supernumerary NBs (from 8 to an average of 16 NBs per brain lobe), without affecting the number of mINPs. Also, they properly show that overexpression of Six4 in a RNAi background successfully rescues the phenotype. Thus, the authors conclude that Six4 is required to prevent supernumerary type II NBs in the larval brain. Then, it is excluded that supernumerary NBs arise from defective asymmetric division, while induction of Six4 RNAi expression in imINPs through an earmuffGal4 driver results in a mild but consistent increase in the number of NBs present (from 8 to 9-10). After, they show that abrogation of Six4 within NBs results in similar levels of PntP1 within NBs and imINPs, but that expression persisted in mINPs, indicating delayed suppression of PntP1. Not only that, but persistent levels of PntP1 promoted by Six4 depletion does not affect levels of Erm. Altogether, this indicates that both Six4 and Erm are involved in PntP1 repression in INPs. In line with the previous finding, overexpression of Six4 within NBs results in type II to type I transformation in 25% of the cases and Pntp1 decreased expression. Also, type II that retained their identity generated fewer INPs. Furthermore, the authors show that, like Erm, Six4 is capable of suppressing PntP1 expression and antagonize the activity of the latter. Hence, by ensuring proper inhibition of PntP1, Six4 is capable of granting imINP to become fate committed. To further elucidate the regulatory role of Six4, the authors show that (i) Six4 is capable of functionally replace Erm to prevent imINP dedifferentiation; (ii) Six4 and Erm independently co-immunoprecipitate with PntP1; (iii) Six4 and Erm coimmunoprecipitate with each other, indicating that these proteins interact with each other. While authors show that Six4 is capable of inhibiting PntP1 during INP maturation, the role of Six4 in NBs is still unclear. While overexpression of Six4 in NBs is capable of inhibiting PntP1, what is its physiological role in NBs? For that, authors co-inhibit PntP1 and Six4 in NBs in hopes that by silencing Six4 it would be possible to restore levels of PntP1 and rescue of the phenotype observed in single PntP1 knockdown (supernumerary Asense-positive NBs and mINP depletion). Nevertheless, the phenotype remained the same in terms of Ase-positive NBs. On the other hand, mINPs were completely depleted in this scenario and the supernumerary phenotype of type II NBs was also inhibited. Nevertheless, the authors show that, at a physiological level, Six4 may in part contribute to the suppression of prospero in newly-generated imINP, such as PntP1 does.
Overall, the authors show great knowledge in terms of the field and the literature available for the full comprehension of this topic. Not only that, but it is possible to observe that the experiments have been well thought out, well-executed, and with a strong rationale to support them. Moreover, the manuscript is well written and can be understood by someone in the field. Due to the nature of the work, the manuscript can be a bit more difficult to follow by a non-specialist. Altogether, these novel results increment the knowledge we have today regarding NBs and INPs homeostasis and contribute greatly to the field.

Point-by-point comments:
(related to Figure 1)
It would be very interesting to also include the cell-type counts per lineage, so the reader has the understanding on whether there are supernumerary NBs within one lineage or if the supernumerary NBs form their independent lineages (from the image, it seems that is the second case). This would corroborate that supernumerary NBs do not form by incorrect asymmetric division, but it would also not be evident that they would form by INP fate-reversion;

Response: As stated in our response to reviewer #1’s comments, we showed in the revised Fig 1 that multiple type II NBs could be observed in single type II NB lineages. We found that about 4% of Six4 knockdown (by pntp1-GAL4) type II NB lineages had multiple type II NBs, while other 96% of NBs formed independent lineages with the number of INPs comparable to that in the WT control. The quantification of the number of mINPs is provided in the revised Fig 1J. Therefore, together with other data we presented, the supernumerary type II NBs are generated by dedifferentiation of imINPs and this happens throughout the development and those generated early during development went on to establish their own independent lineages.

(related to Figure 2)
Possibly, the image could be simplified: maybe just keep the arrows/arrow heads in one of the lineages and/or just keep them in the image with the merged channels;

Response: we have removed most arrows/arrowheads in most figures.

(line 157)
Some CB type I NBs and respective newly-formed GMC and GMCs from the VNC (but not NB) express Six4. This is very interesting in terms of neural stem cell heterogeneity and eventual adult neuronal diversity. Are always the type I NBs and/or GMCs spatially located in the same regions of the brain?

Response: Yes, these Six4-positive type I NB lineages are consistently located in the same regions of the brains. Therefore, as this reviewer indicated, there is heterogeneity even among the type I NBs based on the differential expression of Six4. However, we did not see obvious phenotypes when we knocked down or overexpressed Six4 in type I NB lineages with our limited readout (data not shown). Thus, the function of Six4 in type I NB lineage development remains to be investigated.

(related to Figure 3)
While the experiments are consistently well designed throughout the manuscript, it is unclear what type of control it is used. For instance, in this case (mostly because the phenotype is so subtle), it would be important that the control animals also express two copies of a non-targeting RNAi (like a UAS-mcherry RNAi). Also, in this case, it would be important to know the number of NBs per lineage to understand where the extra NBs formed are located. This would be interesting, considering that the phenotype might arise early in development, during NBs specification (resulting in more NBs and more lineages), or if later during larval stages (resulting in more NBs within one single lineage, which in the representative Figure seems to be the case). It would also be interesting to further dissect the mechanisms of fate reversion by the absence of Six4 through the use of reporter lines (targeting fate-associated proteins) and long-term live cell and/or whole-brain imaging. Figure 3A is missing the scale bar.

Response: 1). We have added an extra negative control in new Fig 3B and 3F by using erm-GAL4(II) to drive expression of two copies of *UAS-mcherry RNAi*. This negative control never showed any extra type II NBs.

2). We quantified the number of type II NBs lineages with multiple type II NBs and found that on average there was one lineage containing multiple type II NBs per brain lobe (text lines 200-202). The supernumerary type II NBs generated from Six4 knockdown in imINPs, together with the new data in Fig S1A-C showing that knocking down Six4 only after larval hatching still resulted in supernumerary type II NBs, demonstrate that the supernumerary type II NBs resulting from Six4 knockdown were generated from dedifferentiation of imINPs rather than defects in NB specification early during development at the embryonic stages.

3). We did try to perform lineage tracing experiments to directly confirm that the supernumerary type II NBs are derived from imINPs, but there were some technical difficulties that made it very challenging to carry out the experiments. Potentially we could use two different strategies for the lineages tracing. One is to drive the expression of UAS-Flippase, which will mediate the excision of the flip-out cassette of act-FRT-STOP-FRT-lacZ, in imINPs while erm-GAL4 is used to drive the expression of UAS-Six4 RNAi in imiNPs. However, since we could only see strong supernumerary type II NBs only when two copies of UAS-Six4 RNAi are used in the erm2/+ background, we had some difficulties to put all the genetic components together needed for the RNAi knockdown and lineage tracing by recombination. Another strategy is to use pntP1-GAL4 to drive the expression of UAS-Six4 RNAi in type II NB lineages and use the LexA-LexAop system to drive the expression of LexAop-Flippase in imINPs. There is an erm-LexA line that could be potentially used for driving the expression of LexAop-Flippase in imINPs. Unfortunately, we found that the erm-LexA line available from the Bloomington stock center does not have the same expression pattern as the erm-GAL4 (II). The erm-LexA is only expressed in a subset of type II NB lineages and it is also expressed in the type II NBs in addition to imINPs in all these lineages, which makes it impossible to express LexAop-Flippase specifically in imINPs for tracing the fates of the daughter cells of imINPs. Due to these technical difficulties, we were not able to successfully carry out the lineage tracing experiments, unfortunately.

4). Scale bar have been added in Fig 3A.

(related to Figure 4)
For simplification, reduce the number of arrows (maybe just keep them in some of the panels);

Response: we have removed some arrows.

(related to Figure 5)
For simplification, reduce the number of arrows (maybe just keep them in some of the panels);

Response: We did as the reviewer suggested.

(line 334)
While it is a strong possibility that Six4 binds directly to Erm, these experiments do not necessarily allow us to conclude that. Instead, it is possible to conclude that both are in close proximity/interact within the same complex/interact within the same chromatin context.

Response: Thanks for the reviewer’s comment. We agree that we cannot completely rule out the possibility that Six4 and Erm are in close proximity/interact within the same complex or interact within the same chromatin context instead of binding with each other directly. Therefore, we have made our statement less strong and included this possibility in our description (lines 355-359). Based on our new results that there is no detectable level of endogenous PntP1 in S2 cells (new S7A-B Fig), we think that interaction between Six4 and Erm is probably not mediated by PntP1. However, the presence of PntP1 likely makes the complex more stable or enhances the binding as coexpression of Flag-PntP1 enhanced the interaction between Six4-HA and Myc-Erm at least at high salt concentration (new S7F-F’ Fig, lane #6-8).

Also, related to Figure 6P, it would be interesting to see whether you would see PntP1 being coIPed with Six4-HA or Myc-Erm, since none of the coIP experiments show that the three proteins are coIPed together: the upper panel shows that when Six4-HA is IPed it brings down Myc-Erm with it and, similarly, when Myc-Erm is IPed, it brings down Six4-HA. In addition, to better understand these questions, it would interesting to test the coIP using higher salt concentrations, to have a better idea how strong of an interaction it is. Also, it would be interesting to further address this question (not necessarily for the scope of this specific paper), like doing chromatinIP or targeted-DamID to address the chromatin binding of Six4/Erm/PntP1, to further dissect this regulatory axis at the molecular level;

Response: in our new S7F Fig (lane #2, 6, 10, and 14), we showed that when Six4-HA, FlagPntP1 and Myc-Erm were coexpressed, these three proteins can be pulled down together, indicating that these three proteins indeed can exist in the same complex. We also performed co-IP with a gradient of salt concentrations from 150mM to 1200mM of NaCl (Fig S7C-E). We could see that the interactions among Flag-PntP1, Six4-HA, and Myc-Erm became progressively weaker with the increase of the salt concentration, but we did not see complete disruption of their interactions even at 1200mM of NaCl, indicating that their interactions are quite strong.

To map the DNA binding profiles of Six4/Erm/PntP1 is definitely an interesting direction to go and is essential for elucidating how these proteins act together to regulate target gene expression as discussed in the discussion part (lines 497-500). We have actually started to work on this and made UAS-PntP1-Dam transgenic line, but as the reviewer commented, it is really beyond the scope of this paper.

(line 334-336)
The authors suggest that inhibition of PntP1 in imINPs putatively occurs via physical interaction with Six4 and Erm. Because this would take place after protein translation, as a post-translational mechanism, it does not justify how transcript levels of PntP1 drastically decrease over lineage progression (unless there is a positive feedback loop where PntP1 regulates its own expression);

Response: Yes, the positive feeback loop is exactly what we thought. Please see lines 490-495 in the discussion.

(related to Figure 6)
Just like previously suggested, for simplification purposes arrows can be removed;

Response: We have reduced the number of arrows.

(line 418-424)
Would it be possible to use an erm-Gal4 in the II chromosome in combination with erm-Gal4 on the III chromosome? Both are expressed in imINPs, but in slightly different stages (either in Ase-negative – R9D11-Gal4 (II) – or in Ase-positive imINPs – R9D11-Gal4 (III)). Maybe this strategy would enlarge the timeframe of expression of the RNAi. Also, the second hypothesis could be easily addressed by using the modERN line expressing Six4-GFP, to see whether induction of the RNAi against Six4 with erm-Gal4 is not enough to decrease levels of Six4 in INPs;

Response: we checked Six4-GFP expression levels in imINPs when erm-GAL4(II) was used to drive two copies of UAS-Six4 RNAi and found that the RNAi knockdown was not efficient enough to dramatically reduce the Six4-GFP levels (new S4D-E Fig). We also combined erm-Gal4(II) and erm-GAL4(III) to drive one copy of UAS-Six4 RNAi, but we did not see any supernumerary type II NBs (new S4A-C Fig).

(line 463 onwards)
Authors discuss whether the presence of Erm and Six4 is required for complex stability. First, while results are very convincing and well curated, I think it is too premature to state that they form a proper complex. It seems too strong of a statement given the results available. From their IP studies, when they express Six4-HA or Myc-Erm, the interaction with PntP1 is of the same extent (also Erm and Six4 are not endogenously expressed in S2 cells, so they cannot compensate each other). By performing a salt gradient, maybe it would be possible to study the stability of this complex. Also, it has been shown that transient overexpression of Insensible in a brat null induces supernumerary type II NBs to synchronously transition into INPs, thus leading to an enrichment of INPs within the brain (Rives-Quinto et al., bioRvix, doi: [https://doi.org/10.1101/2020.02.03.931972](https://urldefense.com/v3/__https://doi.org/10.1101/2020.02.03.931972__;!!GobTDDpD7A!d_k_FbFBnljZW4IBn0R_E__siXJlwMEsKjm15k5tIClL45RUl2s0_4TPPTm_B_0$)). This could be a good strategy to do this same experiment in a Drosophila brain context;
(Related to Supp Figure 1)

Response: As stated above, we have added new data to show that Six4, Erm, and PntP1 could be pulled down in the same complex when they were co-expressed in S2 cells and the interactions at a high salt concentration (1200mM of NaCl) became stronger when all these three proteins were co-expressed (Fig S7F). We also did co-IP with graded concentrations of salt to examine the stability of the interactions among Six4, Erm, and PnP1 and found that their interactions became progressively weaker with the increase of the salt concentration (Fig S7C-E) but were not completely disrupted even at high salt concentrations.

We thank the reviewer for the suggestion to transiently overexpress Insensible in a brat null background to induce supernumerary type II NBs and enrich INPs. That could be very helpful, but due to the time constraint for the revision, we were not able to perform this experiment for our revision.

There is a typo (ventral instead of vental).

Correct the typo in suppl fig 1B and maybe other places.

Response: Thanks for catching the typos. We have corrected all typos we could find.
