# Supplementary material for: Homeodomain protein Six4 prevents the generation of supernumerary Drosophila type II neuroblasts and premature differentiation of intermediate neural progenitors
Source: PLoS Genet. 2021 Feb 8;17(2):e1009371. doi: 10.1371/journal.pgen.1009371 (PMC7895384; doi:10.1371/journal.pgen.1009371)
Supplement: Attachment — Submitted filename: point-to-point responses_resubmission_18Dec2020.docx [file pgen.1009371.s011.docx]

Reviewer #2: I appreciate the authors efforts to address the comments of myself and the other two reviewers. The additional schematic drawings are very helpful. I also appreciate the authors taking the suggestion of two of us and removing many of the arrows and arrowheads from various figures. As I mentioned in my first review, I find the topic of this paper very interesting and it is addressing a very important question – namely how are intermediate neural progenitors prevented from either dedifferentiating or differentiating too quickly. As the authors point out, maintaining the balance of INPs is critical for producing a brain of the right size and cell numbers.

I read the paper several times and I still am having trouble seeing the phenotype just from the images. I have downloaded the original files and while they are of very high resolution it is really difficult for me to see the difference among the different genotypes. In my initial review I had requested that the authors remove the arrows and arrowheads since I wanted to see if I could look at the brain images and see if I came to the same conclusion as the authors. Without the graphs documenting the quantifications, I would not be able to see the difference between many of the panels. For a non-expert it is not clear why some cells are counted while others are not. For example, in panels 2B,C,D the authors place arrows on certain cells. But there are other cells that show the same expression profile that are not marked with arrows. This is true of many of the panels throughout the paper. I think this is going to be problematic for the non-expert and will reduce the enthusiasm for the paper.

Response: We are sorry that the images were still not easy for the reviewer to see the phenotypes. In order to make it easier to the reviewer and readers to see the phenotypes we have made the following changes. 1) We cropped the images for almost all the figures so that we mostly just show the central brain part where type II NBs and type I NBs reside and left the most of the optic lobe part out. In that way, images also become bigger. 2) We showed separated Dpn/E(spl)mg-GFP and Ase staining channels so that readers and reviewers can easily identity the type II NBs, which express Dpn but not Ase and appear as blue in the nucleus in the merged images. We put arrows to point to type II NBs in the separate Dpn and Ase staining channels to assist readers and reviewers to identity the type II NBs. 3) We described in more detail in Fig1 legend how to identify type II NBs. 4) We added dashed lines in most images to demarcate the boundary between the central brain and the optic lobe and pointed out that type II NBs are in the central brain so that readers can know where to identify type II NBs and look at the phenotypes.

One thing that probably makes it a little hard to identify type II NB lineage is that mCD8-GFP/RFP driven by pntP1-GAL4 is sometimes not exclusively expressed in type II NB lineages in the central brain. Depending on genetic background, sometimes it has high expression in glial cells, which makes the signal in type II NB lineages not stand out (e.g. Fig1E). We replaced the image in Fig 1C with an image that shows less mCD8-RFP expression in glial cells. Unfortunately, we were not able to find better images with less expression of mCD8-RFP in the glial cells for other images. Hopefully, with additional Dpn/E(spl)mg-GFP and Ase staining channels, it would be easy now for the reviewer to identify type II NBs and see the phenotypes.

Another thing we would like to point out is that most images we show in the figures are just single focal slices. Therefore, often times not all type II NBs in the larval brains are included in the images. We just chose focal slices that include the majority of the type II NBs or mINPs. Therefore, sometimes there might be some discrepancy of the numbers of type II NBs or mINPs shown in the images and the numbers on the bar graphs.

We have to say that we counted the number of type II NBs and mINPs using the same criteria. All the big cells (about 10-15 mm in diameter) that are labeled with mCD8-GFP/RFP driven by *pntP1-GAL4*, Dpn (or E(Spl)mg-GFP) but not Ase are counted as type II NBs (thus appear as blue in the nucleus in the merged images). Type I NBs are identified as big cells (similar size as type II NBs) that express both Dpn and Ase (thus appear as magenta in the nucleus in the merged images). Whereas mINPs are identified as smaller (about 4-7 mm in diameter) cells that express both Dpn (or E(Spl)mg-GFP) and Ase (so also appear as magenta) as well as mCD8-GFP/RFP driven by *pntP1-GAL4* (if it is included). No cells in the optic lobe are included in our quantification.

We are not sure exactly which figure the reviewer refers to here because there is no Fig 2D. My guess is that the reviewer was referring to the Fig 1B, C, D. The reason why we put arrows for some cells but not others with the same expression profile was because reviewers suggested to remove most arrows and arrowheads. So we just put arrows for some of the cells as an example and leave the readers/reviewers to make the judgement. In any event, we have modified most figures in our revision as described above. We hope that our revised figures are much easier now for reviewers/readers to see the phenotypes unbiasedly.

I think the quantifications that are presented throughout the paper suggest that Six4 is playing an essential role in this maintaining INP balance in numbers. It is just very hard for me to see it without the arrows/arrowheads and charts. I guess I am struggling with understanding how the authors selected some cells and not others even though both cells express the same set of markers.

Response: Please see our response above for how we quantified the number of type II NBs and INPs.

I also feel that some of the images are very small. This was a problem with the first version. Even looking at the high resolution images does not help. It is still very hard to see what is being pointed to in figure 7A,B,F,G,H.

Response: We have enlarged images, particularly those initially shown in the insets. We made those insets as separate panels with the same size as other panels. We also cropped almost all images so that we now just focus on the central brain part as describe above.

The authors mention that Six4 is functioning after larval hatching. Using a tub-GAL80ts, the authors allowed for expression of the RNAi line only after larval hatching and see that each brain lobe had one extra type II NB. This is in contrast to flies that don’t have the tub-GAL80ts, which results in 16 NBs per brain lobe. The authors conclude that since they see one extra NB it must mean that Six4 is functioning after larval hatching. I would think that since most of the extra NBs are not generated when the RNAi line is expressed after larval hatching it would mean that Six4 is actually functioning during embryonic stages.

Response: We are sorry that we did not make it very clear. We did not mean that the supernumerary type II NBs resulting from Six4 knockdown were generated only during larval stages based on this experiment. What we meant was that Six4 knockdown probably did not affect the initial specification of type II NBs from the neuroectoderm during the embryonic stage. The supernumerary type II NBs could be generated during embryonic stages when type II NBs divide to produce INPs. In the revised manuscript, we described that the majority of the supernumerary type II NBs are likely generated during early development at embryonic stages and possibly early larval stages as well and discussed the possible reason why INPs generated during early development might be more susceptible to dedifferentiation in the discussion part (please see the second paragraph of the first section “Knockdown of Six4 results in supernumerary type II NBs” and the first paragraph of the third section “Six4 likely acts in imINPs to prevent dedifferentiation of imINPs” of the “Results” part, and the 3^rd^ paragraph of the “discussion” part).

The manuscript would also be helped if the authors could include a molecular model of how NB progress to imINPs to mINPs to GMCs. There is a lot going on in this pathway and the authors do an excellent job of describing it within the introduction. A diagram would really help the reader.

Response: Thanks for the reviewers’ suggestion. We have included a new diagram in Fig 1B to include some key molecules involved in the development of type II NB lineages.

Reviewer #3: First of all, I appreciate the efforts the authors made to address all the comments, questions, and suggestions proposed by all the reviewers. Overall, while the manuscript was very well written in its initial version, in some instances the figures were a bit difficult to interpret, thus making difficult the understanding of the results. By simplifying and clarifying their meaning, it was clearer what the authors report in the text. Also, in their point-by-point answers to the reviewers, the authors were objective and up-front, addressing most of the questions appropriately. In addition, as suggested by another reviewer, the authors included a much-appreciated schematization of the larval brain, incredibly useful for those that are non-specialists.

I have a number of concerns regarding some of the pivotal findings (some of them serving as the main premise of the paper). For instance, and related to the results depicted in Figure 1, one of the things that was not clear in the initial version of the manuscript, was whether the supernumerary neuroblasts arising upon Six4 depletion were: (i) neuroblast forming independent lineages and those lineages were virtually identical to a wildtype one (thus aligning with the idea that the phenotype promoted by loss of Six4 arises during early NB specification) or; (ii) the ectopic neuroblasts would co-exist withing the same lineage thus supporting the idea of defective asymmetric division (which the authors do not observe) or dedifferentiation/fate reversion of INPs. According to the answer provided by the authors “4% of Six4 knockdown type II NB lineages had multiple type II NBs, while the other 96% of NBs formed independent lineages (…)”. First, if the phenotype is promoted by dedifferentiation of INPs, one would expect lineages with ectopic NBs, that is only verified in 4% of the cases (considering that each lobe has 8 lineages, this means the phenotype is observed in one lineage every 4 lobes or 2 brains). Also, if the NBs are formed at the expense of INPs, one would expect decreased numbers of this cell type (and GMCs) along the affected lineages, which is not really the case (as shown in Figure 1J).

Response: We would like to thank this reviewer for the constructive suggestions below for further revising the manuscript. We really appreciate it. However, we have to say that the results are not necessary contradictory to the dedifferentiation of imINPs. The reason is because if the dedifferentiation of imINPs happens at a low rate (approximately only 1 imINPs per lineage dedifferentiates to adopt the type II NB fate as the total number of type II NBs is only about 16 after Six4 is knocked down) and if the majority of the supernumerary type II NBs are generated during early development at embryonic stages and/or early larval stages as we discussed in the revised manuscript, the majority of the dedifferentiated NBs will be pushed away from their parental NB by their progeny over time and have their own independent set of INPs, GMCs, and neurons. Therefore, most of the supernumerary type II NBs will appear to have their own independent lineages. The average number of mINPs per lineage won’t be significantly different from that in the wild type lineages either because mINPs can only divide about 5-8 rounds and only exist for a short period of time (probably no more than 24 hours – about 8 hours from their birth to becoming mINP plus about 16 hours for 8 rounds of self-renewing divisions if each round takes 2 hours), the mINPs seen in each lineage at any given time are probably those generated within the last 24 hours before brains are dissected for phenotypic analyses, which we usually do at late 3^rd^ instar larval stages (meaning that the lineage has developed for 4-5 days before dissection). A significant reduction in the number of mINPs will be obvious only when the dedifferentiation of imINPs happens more frequently.

Another concern regards the claims that the phenotype takes place after larval hatching, thus excluding that NBs specification during embryonic stages would not be affected. In fact, this was one of the interrogations that I and other reviewers had when looking at the figures provided during the initial submission of the paper. To tackle this problem, the authors controlled the expression of the Six4 RNAi with tubGal80ts, initially rearing the animals at the restrictive temperature and only shifting them to the permissive temperature after larval hatching. In this context, as shown in Figure S1, the authors observed the appearance of one more NB per lobe (in average) in opposition to the 16 NBs they observe when animals without expression of tugGal80ts. Because the phenotype is completely distinct and much stronger in one case versus the other (and while one can argue whether the RNAi is efficient during larval stages), these results indicate that the Six4 is actually exerting its activity during embryonic stages. Having that, and while it is not excluded that Six4 exerts a regulatory role during larva stages, it should not be excluded that the phenotype (supernumerary neuroblasts caused by NB duplication or unfaithful specification during embryonic stages).

Response: As described in our response to the reviewer #2, we did not mean that dedifferentiation only happens after larval hatching when Six4 is knocked down. Sorry we did not make it very clear. In this revised manuscript, we discussed that the supernumerary type II NBs are most likely generated early during development at embryonic stages and possibly early larval stages as well, but the initial specification of type II NBs from the neuroectoderm during the embryonic stages is unlikely to be affected by the expression of UAS-Six4 RNAi driven by pntP1-GAL4. The reason is because like endogenous PntP1 protein, pntP1-GAL4 is only expressed in type II NB lineages but not in the neuroectoderm before type II NBs are specified. Therefore, with pntP1-GAL4 as a driver, we are only knocking down Six4 in type II NBs after they are specified and the specification of type II NBs is unlikely to be affected by Six4 knockdown. Please see the second paragraph of the first section “Knockdown of Six4 results in supernumerary type II NBs” and the first paragraph of the third section “Six4 likely acts in imINPs to prevent dedifferentiation of imINPs” of the “Results” part, and the 3^rd^ paragraph of the discussion part for the detailed explanation.

Later, to investigate whether Six4 generates supernumerary NBs through dedifferentiation of INPs, the authors decide to abolish Six4 levels within INPs and address the number of NBs. Again, as previously, the phenotype is extremely discreet (only 1 to 2 more NBs per lobe, with only 1 type II lineage having more than 1 NB). The phenotype was slightly stronger when Six4 RNAi was performed in erm heterozygous background (Figure 3 E), but still very variable (is most of the cases – 5 in a total of 8 – the phenotype is the same in erm heterozygous background alone, Figure 3D and G). Not only that, but from what is possible to understand from Figure S4 D, E, levels of Six4 within INPs are virtually the same as the control, indicating that the RNAi is not effective when its expression is driven by ermGal4 (in opposition to pointedGal4).

Response: We have to say that variation of phenotypes in individual animals is pretty normal in biological studies. We cannot expect to always see the exact same phenotypes in individual animals and 100% of penetrance of the phenotypes. That’s why we need to do statistical analysis to see if differences are statistically significant.

At this point, I would not advise the execution of complementary experiments. Instead, I think that the text can be reorganized/re-written in order to better reflect the results in a completely unbiased way. Taking that into consideration, I believe that the manuscript is of interest to the field and I recommend its publication if the authors address the critical concerns described previously and described one-by-one bellow:

- I would recommend a change in the title, in order to reflect better the results shown in the manuscript.

Response: Thanks for the suggestion. We have changed the title to more objectively reflect the results.

- From line 128 to 130, the authors claim that “Co-existence of multiple type II NBs in single lineages indicates that these extra type II NBs could be just generated late during larval development and did not have enough time to establish their own independent lineages yet.”. Wouldn’t you expect that, after each round of NB division, the newly formed INP (that usually takes up to 6h to maturate and divide) would fail to commit to its fate and revert back to a NB-like state? If so, shouldn’t there be even more NBs per lobe and per lineage? And why would this occur on such a relatively small scale? Considering that you have 16 NBIIs per lobe upon Six4 depletion, it means that, on average, only one 1 INP per lineage dedifferentiated? Why that only one INP would be affected? Are these newly formed NBs any different from the primary ones? These questions are in the context of INP dedifferentiation upon knockdown of Six4.

Response: We understand the reviewer’s concern, but knocking down a gene involved in promoting INP maturation does not necessary always lead to dedifferentiation of every single imINPs born in every round of division of type II NBs. It depends on whether the gene is absolutely required for maturation of imINPs or partially dispensable. When multiple genes function in the same pathway, not every gene is equally important. Some might be more critical than others. Furthermore, if loss-of-function phenotypic analyses are carried out by RNAi knockdown, the severity of the phenotype also depends on the RNAi knockdown efficiency. One example is that as shown in Fig 6F and J, knockdown of Erm at different temperatures led to different degrees of severity of the phenotypes. Erm has been well documented for its role in preventing dedifferentiation of imINPs. Yet, when knockdown of Erm was done at 25^o^C, the phenotype was weak and there were only about total 15 type II NBs in one brain lobe, similar to Six4 knockdown. Most of these supernumerary type II NBs established independent lineages with their own set of INPs and GMCs as observed in Six4 knockdown brains. When Erm was knocked down at 29^o^C, the supernumerary type II NB phenotype became more severe, with about 100 type II NBs per brain lobe. However, even with this severity, many mature INPs were still generated and associated with individual type II NBs, indicating that when Erm was knocked down at 29^o^C, most imINPs can still differentiate to become mature INPs instead of dedifferentiating back to the NB fate (if calculated based on the total number of type II NBs, probably only 12-13 imINPs dedifferentiate to become type II NBs in each lineage, but a single type II NB could probably generate a total of 50-60 imINPs in 4-5 days of development if the NB divide once every 2 hours).

We do agree with the reviewer that the supernumerary type II NB phenotype resulting from Six4 knockdown is relatively weak. But that is what we observed. The weak phenotype could be that a small amount of endogenous Six4 protein below the detectable level may still exist after Six4 knockdown or that Six4 functions partial redundantly with other genes such as Erm (as we demonstrated that Erm Six4 double knockdown gave synergistic enhancement of the phenotype). Or simply Six4 may not be as critical as other genes in preventing dedifferentiation of imINPs.

We do not know whether there is any difference between the dedifferentiated type II NBs resulting from Six4 knockdown and parental type II NBs at the molecular level. That will require comparison of gene expression profiles between them and more phenotypic analyses, which is really beyond the scope of this study. But according to other published studies (e.g. Narbonne-Reveau K. et al, Elife 2016, doi: [10.7554/eLife.13463](https://doi.org/10.7554/eLife.13463)), certainly there are some differences between the dedifferentiated type II NB and wild type type II NBs. For example, the dedifferentiated type II NBs (at least a subset) will not differentiate and terminate the self-renewal during the early pupal stage, whereas wild type type II NBs will undergo differentiation and disappear during metamorphosis. Some dedifferentiated NBs may also continue to express early temporal identity genes at late developmental stages or even at adult stages.

- From line 154 to 156 the authors conclude that knockdown of Six4 during larval stages (using tubgal80ts) supports that the supernumerary neuroblasts arise during larval development and not during NB specification. Unfortunately, these results do not necessarily support that statement. In no place in the paper should be excluded the embryonic contribution of Six4 (in fact, Six4 could play two distinct roles during animal development: (i) initially through regulation of NB specification and (ii) later in the maintenance of INP pool). This should be clearly stated in the results section and, if appropriate, the authors could develop on this in the discussion section.

Response: As described above, we discussed that initial specification of type II NBs from the neuroectoderm during the embryonic stages is unlikely to be affected by the expression of *UAS-Six4 RNAi* driven by *pntP1-GAL4*. The reason is because *pntP1-GAL4* is only expressed in type II NBs but not in the neuroectoderm before type II NBs are specified. Therefore, with *pntP1-GAL4* as a driver, we are only knocking down Six4 in type II NBs after they are specified. Six4 knockdown unlikely affects the initial specification of type II NBs from the ectoderm during the embryonic stages.

However, we do agree that the majority of the supernumerary type II NBs resulting from Six4 knockdown could be generated during early development including embryonic stages when type II NBs also divide to produce INPs. We also discussed the possible reason why the majority of the supernumerary type II NBs are likely generated during early development in the Discussion part (the 3^rd^ paragraph).

- From line 204 to 205 the authors conclude that “Although the phenotype is weak, the consistent generation of extra type II NBs and the existence of multiple type II NBs in single lineages indicate that the supernumerary type II NB phenotype likely results from dedifferentiation of imINPs.”. This is clearly an overstatement and the sentence/section should be changed in order to factually report what is observed. From the image provided, it is not clear the co-existence of 2 or more NBIIs per lineage and, indeed, the phenotype is very weak (likely due to the inefficiency of the RNAi in INPs). At this point, and without any further experiments, it is difficult to say that, by itself, abrogation of Six4 leads to dedifferentiation of INPs (you would require further experimentation to appropriately test this).

Response: 1) We are sorry that we did not make it very clear how we determine whether or not multiple NBs are in the same lineage. Our judgement is based on whether the multiple NBs are in the same cluster of cells with only one axonal track not their physical closeness. The dedifferentiated type II NBs may not always sit next to the parental NB because overtime the dedifferentiated NBs will be pushed away from the parental NB by their progeny born after the dedifferentiated NBs are generated unless the dedifferentiated NBs are just generated before brain dissection. In any event, we have replaced the image for Fig 3C with a new one, which shows two type II NBs sit next to each other in a single lineage.

2) We have revised our wording as follows “the consistent generation of extra type II NBs and the existence of multiple type II NBs in single lineages indicate that imINPs could dedifferentiate to adopt the type II NB fate when Six4 was knocked down in imINPs.” We also added the following sentence at the end of the second paragraph of the “Discussion” part: “However, to provide definitive evidence to demonstrate that the imINPs dedifferentiate type II NB fate, it will be essential to perform a lineage tracing experiment to follow the fate of the progeny of imINPs. Unfortunately, we were not able to perform the experiment due to some technical difficulties”. I hope that the reviewer would agree this statement is more acceptable”.

- From line 207 to 210. I do think that this result is more indicative (but not conclusive) regarding the role of Six4 in INP dedifferentiation. Since the idea is that ectopic NBs are formed at the expense of INPs, it would be interesting to determine the number of mINP in this specific experiment (considering that you have staining for both Ase and Dpn, this should be fairly simple to do).

Response: We have quantified the number of mINPs as suggested and add a bar graph in the new Fig. 3H.

- When pntp1 and erm start to be introduced in the results section (namely regarding synergistic role with Six4), it would be interesting to include a scheme depicting the molecular pathway through which a type II lineage progresses (NB to imINP, the maturation process of the INP and its asymmetric division to give rise to GMCs).

Response: Thanks for the suggestion. Certainly a diagram will be very helpful for the readers. We have added a new diagram in Fig 1B and cited Fig 1B when we introduced the function of Erm (line 221) and PntP1 (line 408) in the results part.
